# Human Milk Oligosaccharides and Bacterial Profile Modulate Infant Body Composition during Exclusive Breastfeeding

**DOI:** 10.3390/ijms23052865

**Published:** 2022-03-05

**Authors:** Ali S. Cheema, Zoya Gridneva, Annalee J. Furst, Ana S. Roman, Michelle L. Trevenen, Berwin A. Turlach, Ching T. Lai, Lisa F. Stinson, Lars Bode, Matthew S. Payne, Donna T. Geddes

**Affiliations:** 1 School of Molecular Sciences, The University of Western Australia, Crawley, WA 6009, Australia; alisadiq.cheema@research.uwa.edu.au (A.S.C.); zoya.gridneva@uwa.edu.au (Z.G.); ching-tat.lai@uwa.edu.au (C.T.L.); lisa.stinson@uwa.edu.au (L.F.S.); 2 Larsson-Rosenquist Foundation Mother-Milk-Infant Center of Research Excellence, University of California San Diego, La Jolla, CA 92093, USA; aloeffler@health.ucsd.edu (A.J.F.); acroman@sdsu.edu (A.S.R.); lbode@health.ucsd.edu (L.B.); 3 Department of Pediatrics, University of California San Diego, La Jolla, CA 92093, USA; 4 Centre for Applied Statistics, The University of Western Australia, Crawley, WA 6009, Australia; michelle.trevenen@uwa.edu.au (M.L.T.); berwin.turlach@uwa.edu.au (B.A.T.); 5 Division of Obstetrics and Gynaecology, School of Medicine, The University of Western Australia, Subiaco, WA 6008, Australia; matthew.payne@uwa.edu.au; 6 Women and Infants Research Foundation, Subiaco, WA 6008, Australia

**Keywords:** human milk, human milk oligosaccharides, human milk bacteria, microbiome, 16S rRNA gene, breastfeeding, infant, mother, body composition, bioelectrical impedance spectroscopy, intake, concentration, lactation

## Abstract

Human milk is a complex and variable ecosystem fundamental to the development of newborns. This study aimed to investigate relationships between human milk oligosaccharides (HMO) and human milk bacterial profiles and infant body composition. Human milk samples (*n* = 60) were collected at two months postpartum. Infant and maternal body composition was measured with bioimpedance spectroscopy. Human milk bacterial profiles were assessed using full-length 16S rRNA gene sequencing and 19 HMOs were quantitated using high-performance liquid chromatography. Relative abundance of human milk bacterial taxa were significantly associated with concentrations of several fucosylated and sialylated HMOs. Individual human milk bacteria and HMO intakes and concentrations were also significantly associated with infant anthropometry, fat-free mass, and adiposity. Furthermore, when data were stratified based on maternal secretor status, some of these relationships differed significantly among infants born to secretor vs non-secretor mothers. In conclusion, in this pilot study the human milk bacterial profile and HMO intakes and concentrations were significantly associated with infant body composition, with associations modified by secretor status. Future research designed to increase the understanding of the mechanisms by which HMO and human milk bacteria modulate infant body composition should include intakes in addition to concentrations.

## 1. Introduction

Exclusive breastfeeding during the first six months of life delivers a plethora of short- and long-term health benefits [1,2]. Human milk provides a constant supply of bacterial microbiota and bioactive molecules, such as human milk oligosaccharides (HMOs), to the infant during early life, contributing to intestinal homeostasis, immune development, and protection against infection [2,3,4,5]. The microbial and bioactive constituents of human milk may also influence the development of infant body composition [6,7,8], which in the early months postpartum is known to play a significant role in the programming of health later in life [9,10].

HMOs are the third most abundant solid component of human milk, representing about 20% of human milk carbohydrates [11]. HMOs play various functional roles in infant development (4–7). They serve as prebiotics for beneficial infant gut bacteria [7,12], exert immunomodulatory effects, and selectively reduce colonisation of the gut epithelium by certain pathogens, thereby decreasing disease incidence in early life [7]. HMO composition varies between mothers [13] and over the course of lactation [14], and is related to maternal characteristics, environmental exposures, and breastfeeding exclusivity [13,15]. Studies investigating associations between HMOs and maternal adiposity have reported conflicting results, showing both positive and negative correlations between maternal adiposity and individual/total HMO concentrations [15,16,17,18,19], or in contrast no associations [13,20]. These previous studies have used weight and body mass index (BMI) as a proxy measure for adiposity despite BMI being an unreliable measurement of body composition [21]. Such divergent results highlight the need to further examine the influence of maternal characteristics and body composition on HMO profiles.

Numerous studies have investigated the influence of human milk bacteria and HMOs on infant faecal bacterial profiles [22,23,24,25,26,27]; however, few have examined associations between human milk bacteria and HMO composition. Some studies have observed positive correlations between total HMO concentration and the relative abundance of human milk bacteria, including *Bifidobacterium* spp. and *Staphylococcus* spp. [28,29,30,31]. Additionally, both positive and negative associations between individual HMOs and specific human milk bacteria have been reported [28,32,33,34]. To further complicate interpretation, there is little overlap between previous studies in regard to the HMOs and human milk bacteria included in the analyses, inhibiting collective conclusions. Such work has also not been conducted in an Australian population.

Associations between HMO composition and infant growth parameters have been reported, with few studies measuring infant body composition. Total HMO concentration was positively associated with greater infant fat-free mass (FFM) and fat-free mass index (FFMI) and decreased fat mass (FM) index (FMI), %FM, and FM to FFM ratio (FM/FFM) [9], whilst in another study higher HMO diversity and evenness at one month postpartum were associated with lower total FM and %FM [6]. Studies investigating individual HMOs have also reported differing associations [6,8,35,36,37,38]. For example, sialyllacto-N-tetraose c (LSTc), has been positively correlated with weight-for-length *z*-score (WLZ) [37], but not weight-for-age *z*-score (WAZ) [38]. Similarly, one study reported that lacto-N-fucosylpentose I (LNFP I) had no relationship with infant growth over the first four months of life [37], while another study showed that higher concentration of LNFP I was associated with lower infant weight, lean mass, and FM [6].

Infant human milk intake has also long been known to influence the breastfed infants growth [39], but little is known about the impact of HMO intake on infant body composition. Infant intakes of specific HMOs have been positively associated with infant growth over the first six months of life [19], while total HMO intake has not been associated with infant growth or adiposity [9]. These differing results are in the same vein as those seen between HMOs and maternal adiposity/body composition, and provide the impetus for further investigation.

Given the inconclusive research into the effect of HMO and human milk bacterial profiles on infant growth and body composition, it is possible that the study design and the population studied has contributed to these. This study seeks to address some of these limitations by focusing on healthy exclusive breastfeeding dyads, negating the need to account for the major confounder of complementary foods and/or formula consumption. Additionally, to measure infant body composition we used bioelectrical impedance spectroscopy, which has been shown to improve the prediction of fat-free mass compared to anthropometry alone and reduce the bias [40,41]. In addition, a more holistic approach is important to integrate multiple factors into the design to elucidate interactions between human milk components rather than examining them in isolation. Furthermore, measurement of HMO intakes will improve conclusions by addressing the amount of specific HMOs consumed rather than relying on concentrations alone that have been found to be misleading [9].

The aim of this study was to investigate associations between HMOs and the predominant bacterial genera present in human milk and their relationships with the body composition of healthy term three-month-old exclusively breastfed infants. In addition, associations between maternal body composition and HMO and bacterial profiles were explored.

## 2. Results

### 2.1. Participants Characteristics, Anthropometrics and Body Composition

Sixty-seven dyads were recruited; seven were excluded due to formula use (*n* = 2), absence of body composition and 24-h milk intake measurements (*n* = 1) and low 24-h milk intake (<500 mL/day; *n* = 4). Missing data included maternal weight, BMI, and body composition measurements (*n* = 5), infant weight and length measurements, WLZ, WAZ, length-for-age *z*-score (LAZ), BMI and BMI-for-age *z*-scores (BMIAZ) (*n* = 5), infant body composition measurements (*n* = 7), infant head circumference and head circumference-for-age *z*-scores (HCAZ) (*n* = 10), and 24-h milk intake and intakes of HMOs (*n* = 13). Out of 60 mothers, 49 were identified as secretors and 11 as non-secretors, and their characteristics are shown in Table S1. There were no significant differences in infant and maternal characteristics between secretor and non-secretor mothers. Maternal and infant body composition measurements and infant anthropometric *z*-scores at 3 months postpartum are shown in Table S2.

### 2.2. Human Milk Bacterial Composition

**Figure 1:**
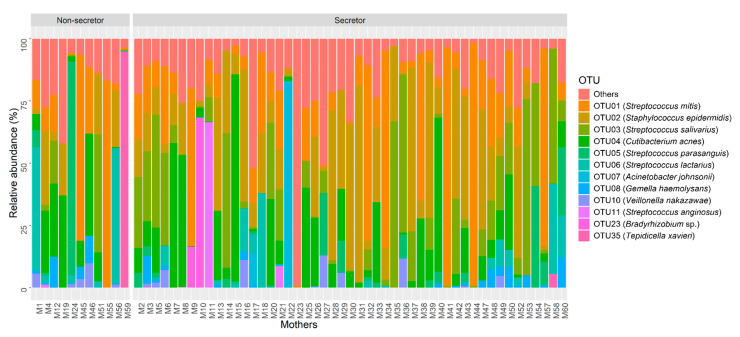
Relative abundance of the 12 most abundant OTUs in human milk samples based on maternal secretor status (non-secretor, *n* = 11; secretor, *n* = 49).

The most abundant operational taxonomic units (OTUs) in human milk mapped to *Streptococcus mitis*, *Staphylococcus epidermidis*, *Streptococcus salivarius* and *Cutibacterium acnes*. In total, 12 OTUs represented ≥1% of the bacterial community observed across all samples (Figure 1). OTUs and associated bacterial genera representing <1% relative abundance are combined in the “other” category and presented in Table S3.

### 2.3. Human Milk Oligosaccharides

**Figure 2:**
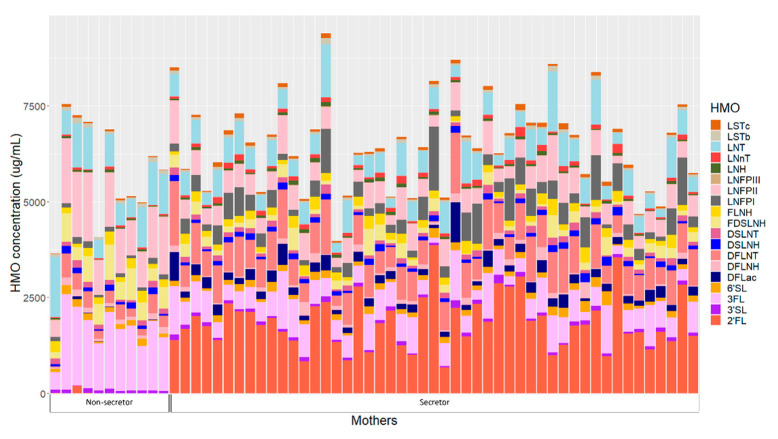
Human milk oligosaccharide concentrations in individual mothers based on secretor status. 2′FL—2′-fucosyllactose; 3′SL—3′-sialyllactose; 3FL—3-fucosyllactose; 6′SL—6′-sialyllactose; DFLac—difucosyllactose; DFLNH—difucosyllacto-N-hexaose; DFLNT—difucosyllacto-N-tetrose; DSLNH—disialyllacto-N-hexaose; DSLNT—disialyllacto-N-tetraose; FDSLNH—fucodisialyllacto-N-hexaose; FLNH—fucosyllacto-N-hexaose; LNFP I—lacto-N-fucopentaose; LNFP II—lacto-N-fucopentaose II; LNFP III—lacto-N-fucopentaose III; LNH—lacto-N-hexaose; LNnT—lacto-N-neotetraose; LNT—lacto-N-tetrose; LSTb—sialyl-lacto-N-tetraose b; LSTc—sialyl-lacto-N-tetraose c.

HMOs concentrations of individual mothers are presented in Figure 2. Human milk samples were dominated by five HMOs: 2′-fucosyllactose (2′FL), 3-fucosyllactose (3FL), lacto-N-tetrose (LNT), lacto-N-fucopentaose II (LNFP II) and difucosyllacto-N-tetrose (DFLNT) (Table S4). Secretor mothers had significantly higher concentrations of 2′FL, difucosyllactose (DFLac), lacto-N-neotetraose (LNnT), lacto-N-fucopentaose I (LNFP I), sialyl-lacto-N-tetraose c (LSTc) and DFLNT, while non-secretors had higher concentrations of 3FL, LNT, LNFPII, sialyl-lacto-N-tetraose (LSTb), difucosyllacto-N-hexaose (DFLNH) and fucodisialyllacto-N-hexaose (FDSLNH) (Table S4).

Twenty-four hour intakes of HMOs differed between infants born to secretor and non-secretor mothers (Table S5). Infants born to mothers with secretor status had significantly higher intakes of 2′FL, DFLac, LNnT, LNFP I, LSTc, DFLNT and lacto-N-hexaose (LNH), while infants born to non-secretors had higher 3FL, LSTb, LNFPII, DFLNH and FDSLNH intakes.

### 2.4. Maternal Characteristics and Human Milk Oligosaccharides

**Figure 3:**
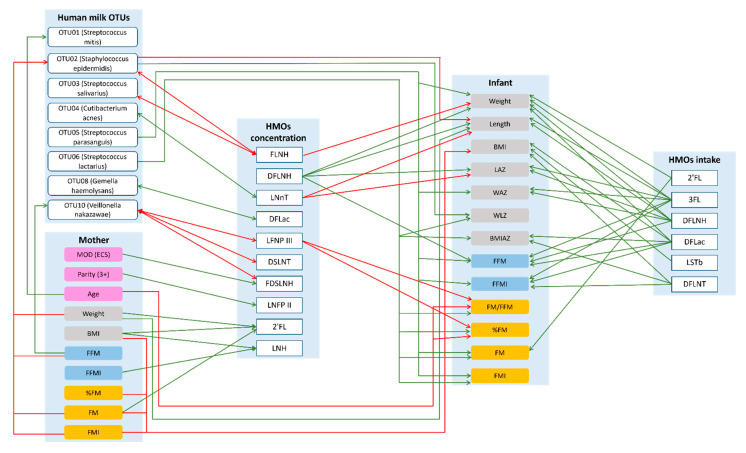
Possible lactocrine programming of the infant body composition irrespective of maternal secretor status. Green arrows indicate positive and red arrows indicate negative associations. 2′—2′-fucosyllactose; 3FL—3-fucosyllactose; BMI—body mass index; BMIAZ—BMI-for-age *z*-scores; DFLac—difucosyllactose; DFLNH—difucosyllacto-N-hexaose; DFLNT—difucosyllacto-N-tetrose; DSLNT—disialyllacto-N-tetraose; ECS—emergency cesarean section; FDSLNH—fucodisialyllacto-N-hexaose; FFM—fat-free mass; FFMI—fat-free mass index; FLNH—fucosyllacto-N-hexaose; FM—fat mass; FMI—fat mass index; LAZ—length-for-age *z*-score; LNFP II—lacto-N-fucopentaose II; LNFP III—lacto-N-fucopentaose III; LNH—lacto-N-hexaose; LNnT—lacto-N-neotetraose; LSTb—sialyl-lacto-N-tetraose b; MOD—mode of delivery; WAZ—weight-for-age *z*-score; WLZ—weight-for-length *z*-score.

Maternal anthropometry and body composition were positively associated with 2′FL and lacto-N-hexaose (LNH) concentrations (Table 1, Figure 3). However, the associations differed depending on maternal secretor status. In secretor mothers, adiposity measures were positively associated with the concentration of 2′FL and fucosyllacto-N-hexaose (FLNH), while in non-secretors, positive associations were observed for 2′FL and FLNH and negative with 3′-sialyllactose (3′SL) (Table 1). Regardless of secretor status, parity and emergency caesarean section were positively associated with LNFP II and FDSLNH, respectively, while in non-secretor mothers, parity was negatively associated with lacto-N-fucopentaose III (LNFP III) (Table S6).

### 2.5. Maternal Characteristics and Human Milk Bacterial Profile

Maternal weight, FFM, FM and FMI were negatively associated with the relative abundance of *S. epidermidis* and FFM was positively associated with the relative abundance of *Veillonella nakazawae*, whilst maternal age was positively associated with the relative abundance of *S. mitis*, regardless of maternal secretor status (Table 2, Figure 3). However, the relationship between some maternal characteristics/body composition and OTU relative abundances differed depending on maternal secretor status. In secretor mothers, anthropometry and adiposity were negatively associated with the relative abundance of *S. salivarius* and FFM was positively associated with relative abundance of *V. nakazawae*. While in non-secretors, maternal anthropometry and body composition measures were positively associated with relative abundances of *Gemella haemolysans* and *V. nakazawae* and maternal age with *Streptococcus lactarius* (Table 2). Additionally, in secretor mothers, emergency caesarean section was positively associated with increased relative abundance of *S. parasanguis* (Table S6).

### 2.6. Maternal Characteristics and Infant Body Composition

Maternal characteristics had both positive and negative associations with infant anthropometry and body composition (Table 3, Figure 3) and differed depending on maternal secretor status. In secretor mothers, maternal FFM was positively associated with infant length. While in non-secretors, associations between maternal FFM parameters and infant body composition were negative. Additionally, in non-secretor mothers emergency caesarean section was associated with increased infant weight, adiposity and FFM (Table S6).

### 2.7. Human Milk Oligosaccharide Concentration and Human Milk Bacterial Profile

Having a higher concentration of FLNH was associated with lower relative abundances of *S. epidermidis* and *S. salivarius*, while higher concentrations of LNFP III, disialyllacto-N-tetraose (DSLNT), FDSLNH were associated with lower relative abundance of *V. nakazawae*. Higher concentrations of LNnT and DFLac were associated with increased relative abundances of *C. acnes* and *G. haemolysans* (Table 4, Figure 3) and differed depending on maternal secretor status. In secretor mothers, negative associations were observed between 3′SL and *S. epidermidis* and DFLac and *V. nakazawae*. While for non-secretor mothers, both positive and negative associations were observed between HMOs and bacterial OTUs (Table 4).

### 2.8. Human Milk Bacterial Profile and Infant Body Composition

After adjusting for 24-h milk intake, greater relative abundances of three OTUs (*S. epidermidis*, *S. parasanguis* and *S. lactarius*) were associated with increases in infant anthropometry, adiposity and FFM except for a negative association between *S. epidermidis* and infant length (Table 5, Figure 3), regardless of secretor status. There were associations which differed between infants born to secretor and non-secretor mothers when the interaction term was present in the final model. In infants born to non-secretors, the relative abundance of *S. mitis* was negatively associated with anthropometry, while *S. parasanguis* was positively associated with BMIAZ (Table 5). No associations were observed for infants born to secretor mothers.

### 2.9. Human Milk Oligosaccharide Concentrations and Infant Body Composition

Regardless of maternal secretor status, higher concentrations of FLNH, LNnT and LNFP III were negatively associated with infant anthropometry and body composition measures, while DFLNH was positively associated (Table 6, Figure 3). However, some associations differed depending on secretor status. In infants of secretor mothers, 3′SL concentration was positively associated with FFM. In infants of non-secretors, the concentrations of DFLNT were positively associated and FLNH was negatively associated with the anthropometry and body composition parameters (Table 6).

### 2.10. Human Milk Oligosaccharide Intake and Infant Body Composition

Intakes of 2′FL, 3FL, DFLac, DFLNH, DFLNT and LSTb were positively associated with infant body composition measures, regardless of maternal secretor status (Table 7, Figure 3). However, secretor status-dependent associations were also observed. In infants born to secretor mothers, 3′SL intake was positively associated with body composition. While in infants born to non-secretor mothers, intakes of 6′-sialyllactose (6′SL) and FDSLNH were negatively associated with body composition (Table 7).

## 3. Discussion

**Figure 4:**
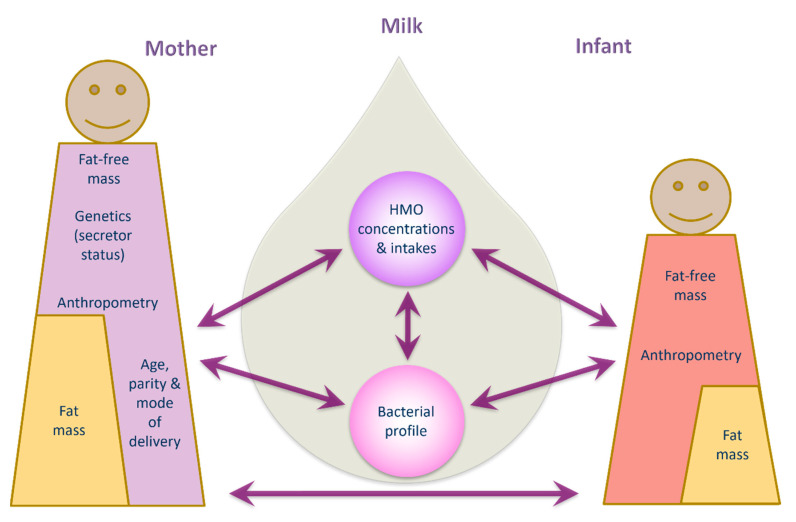
Potential interconnecting pathways of lactocrine programming of infant body composition.

Human milk is a highly complex matrix containing bacterial communities and biologically active molecules that are fundamental for infant development [15,42,43]. This study sheds new light on relationships between human milk bacteria and HMOs and documents associations between human milk components and maternal body composition, and infant growth and body composition development during the exclusive breastfeeding period. Data presented herein suggest potential influential pathways from mother to infant, based on multiple associations between maternal characteristics/body composition and HMO, human milk bacterial profiles and infant body composition (Figure 4).

Maternal factors including genetics, age, ethnicity, and pre-pregnancy BMI are known to influence HMO composition [8,13,16,44,45]. We have also demonstrated relationships between maternal body composition and HMO concentrations during the exclusive breastfeeding period. We observed positive associations between maternal weight, BMI, and body composition measures (FM, FFMI) and concentrations of 2′FL and LNH. McGuire et al. also found maternal weight and BMI to be positively correlated with 2′FL and FLNH, respectively, but not LNH. They found further positive associations between weight and LNFP III and DFLNT and negative associations of weight and BMI with LNnT and DSLNT [15]. Although the McGuire et al. study had 410 participants, they were from eleven international sites and human milk samples were collected between two weeks and five months postpartum. The differences between studies suggests that lactation stage and geographical location have significant impacts on HMO profiles and concentrations [13,15]. However, it remains to be seen whether changes in maternal body composition during lactation modulate concentrations of HMOs and should be studied in a longitudinal manner to account for the stage of lactation. These studies will provide the rationale for maternal interventions designed to impact infant growth and development.

Furthermore, based on maternal secretor status, we found in secretor women that weight and adiposity (BMI, %FM, FM and FMI) were positively associated with concentrations of 2′FL and FLNH. 2′FL as well as DFLac have been positively related to maternal pre-pregnancy BMI only in secretor mothers [35,46], with no data for body composition during lactation. Associations differed in non-secretors in our study, similar to another study that showed that maternal BMI was negatively associated with 3′SL [19]. Interestingly, third trimester maternal fasting plasma glucose and insulin levels have been negatively associated with total HMO, 3′SL and DSLNT concentrations in non-secretors, while in secretor mothers, DFLac and LNFP II concentrations increased and LSTb and LSTc decreased as insulin sensitivity increased at two months postpartum [47]. This is of interest, as we have previously shown that maternal BMI and FMI are positively associated with human milk insulin concentrations [48], and therefore we speculate that maternal body composition may influence plasma insulin levels, which may regulate insulin signaling in the mammary epithelium and thereby potentially play a role in modulating HMO synthesis. However, future studies will be required to investigate HMO synthesis and the factors impacting HMO profiles.

The mode of delivery has previously been related to human milk macronutrient and bacterial profiles [49,50,51,52,53,54], but data on HMOs are sparse. We found higher concentrations of FDSLNH in milk of women who had an emergency caesarean section. However, we did not confirm lower levels of 3′SL, 2′FL and 6′ galactosyllactose in women delivering by caesarean section [55], potentially due to the differences in populations and geographical location. However, lack of labour-associated paracrine and autocrine factors [56] including different patterns of maternal cortisol release during elective caesarean section [57] may be implicated in HMO composition in addition to the increased likelihood of delayed secretory activation or the initiation of breastfeeding.

In our study, parity was positively associated with LNFP II concentration. Ferreira et al., reported correlations between parity and LNFP II, DFLNT, LNH and FDSLNH at days 2–8 [16], while Samuel et al, reported lower concentrations of LNnT and higher concentrations of LNFP II and LNFP V at day 17 [55]. Others have found higher levels of LNnT, LNT and lower levels of 3FL and 2′FL with increased parity [13,18]. Ferreira et al. also reported that different HMOs contributed to the HMO profile based on parity using non-negative matrix factorization [16]. As parity is associated with maternal BMI [58,59] and increased interpregnancy BMI with every additional delivery [58], the relationship between maternal BMI and human milk fatty acid composition [60] and fat and protein concentration [61] indicate that it is possible that parity may impact HMO content. Furthermore, shorter and longer interpregnancy intervals have been shown to influence maternal body composition and breastfeeding practices [62], which may impact HM composition, including HMOs.

Maternal BMI has been related to human milk bacterial genera [51,63]. A novel finding of this study is that maternal FFM and adiposity were associated with the relative abundances of *S. salivarius*, *S. lactarius*, *G. haemolysans*, *V. nakazawae*, *S. mitis*, and *S. epidermidis*, and that these relationships changed with maternal secretor status. Given that human milk bacteria, including *S. epidermidis*, *Streptococcus* spp. and *Veillonella* spp., are among the early colonizers of the infant gut [31,64], they may have important implications for infants. *S. epidermidis* is an opportunistic pathogen and common infant gut colonizer and has been shown to be associated with sporadic diarrhoea in children [65]. Furthermore, a high abundance of *S. epidermidis* has been associated with allergic diseases including food allergy [66], increased risk of atopic eczema [67] and obesity [68], and may participate in modulation of the development of the neonatal immune system [66]. *Streptococcus* spp. and *Veillonella* spp., frequently co-occur [69]; *Streptococcus* spp. ferments carbohydrates, yielding lactic acid as their predominant fermentation end product [70], while *Veillonella* spp. utilizes lactic acid as a carbon and energy source [71], thus they are likely involved in maintenance of the intestinal microbiota.

In our study, maternal factors including body composition are related to human milk microbiota and HMO composition. The mechanisms involved are not established, however, it is feasible that diet regulates maternal body composition [72,73], which subsequently influences human milk microbiota and HMO composition. This raises the potential for maternal interventions to improve maternal body composition and health through diet, thereby influencing milk microbiota and HMO interactions to improve infant growth and development both directly and indirectly (through the infant gut microbiome).

Our study indicates that individual HMO concentrations may influence human milk bacterial profiles during the exclusive breastfeeding period. We identified positive and negative associations between individual HMO concentrations and the relative abundance of *S. epidermidis*, *S. salivarius*, *C. acnes*, *G. haemolysans* and *V. nakazawae*. These relationships are plausible, as in vitro studies have shown a stimulatory effect of HMOs on *Staphylococcus* sp. [30], *Bacteroides* sp. [74], and *Bifidobacterium* sp. [75], and inhibition of group B *Streptococcus* [76]. However, our associations differ from previous studies, with others documenting relationships between 2′FL, DSLNT, disialyllacto-N-hexaose (DSLNH), 3′SL, LNFP II, LNFP III, LSTc and 6′SL and multiple bacterial genera including *Staphylococcus* sp., *Bifidobacterium* sp., *Ralstonia* sp., *Novosphingobium* sp., *Atopobium* sp., *Leptotrichia* sp., *Veillonella* sp. and *Porphyromonas* sp. [28,30,31,32,34,46]. The differences may be due to factors such as methodology, in particular, sequencing different regions of the 16S rRNA gene, environmental factors known to impact HMOs and the human milk microbiota, or differences in geographical location [15,77,78,79,80].

Our study extends prior findings by identifying relationships between HMOs and bacteria that were dependent upon maternal secretor status. For instance, in non-secretor mothers, several HMOs were negatively associated with four of the most abundant human milk OTUs: *S. parasanguis*, *S. lactarius*, *G. haemolysans* and *C. acnes*. A negative relationship between total HMO concentration and *Corynebacterium* sp., LNnT and *Streptococcus* sp. and a positive relationship between 3′SL and *Streptococcus* sp. has also been observed in another study [46], whilst none have been reported for *S. lactarius* and *G. haemolysans*. In our secretor mothers, 3′SL was negatively associated with *S. epidermidis*. Additionally, *S. epidermidis* was also negatively associated with infant length and positively with WLZ. Williams et al. reported a positive association between total HMOs and 2′FL and the relative abundance of *Staphylococcus* sp. [28], and Moossavi et al. identified positive relationships between the sialylated HMOs: 3′SL, 6′SL, LSTb, LSTc, DSLNT and DSLNH and *Staphylococcus* spp. [32]. We speculate that increased 3′SL being related to decreased *S. epidermidis* may play an important role in reducing the risk of sepsis in infants [81] and reducing maternal risk of mastitis by reducing the growth of *Streptococcus* spp. to outcompete the pathogenic strains of *Staphylococcus* spp. [80]. Additionally, HMOs are prebiotic agents that serve as metabolic substrates, modulate the immune system, and inhibit pathogen-host cell interactions [82]. Indeed, Bifidobacteria and *Bacteroides* sp. were found to only be present in one-month-old breastfed infant faecal microbiota if 2′FL and LNFP was present in the milk they consumed [83], confirming that HMOs are a driver of the bacterial profile. However, there is still a need to elucidate the mechanisms by which HMOs modulate human milk bacteria and to understand the significance of oral and skin bacteria with respect to infant outcomes.

Another novel finding of the current study was the association between human milk OTUs and infant body composition. The relative abundance of *S. parasanguis* and *S. lactarius* was positively associated with infant FFM and FM. Additionally, in infants born to non-secretor mothers, the increased relative abundance of *S. parasanguis* was positively associated with infant BMIAZ, while *S. mitis* was negatively associated with head circumference, LAZ and HCAZ. These results suggest that human milk bacteria may be involved in modulating infant growth and body composition, potentially via colonisation of the infant intestinal tract [84,85]. If this holds true then this represents a way to modulate human milk microbiota through maternal diet to shape infant gut microbiota [86] and to regulate infant body composition development.

In this study we also provide further support for the hypothesis that HMOs are related to infant growth, potentially directly and/or mediated by the infant gut microbiome [7]. We observed a positive relationship of DFLNH concentration with infant weight, length, LAZ, and FFM, while FLNH, LNnT and LNFP III were negatively associated with infant adiposity and anthropometry measures (weight, length, LAZ, %FM, and FM/FFM). These associations differ from previous studies, with others documenting negative associations between LNnT and HAZ [36], between LNnT and %FM at six months [6], and between LSTc and infant weight, length [18], and WAZ score [38]. Studies that analysed only secretor mothers report differing results, including positive associations of DFLNH with change in LAZ and LNFP I + III with change in WLZ between 6–12 months [87], negative associations of LNnT with height and weight *z*-scores in the first 12 months [35], and negative associations of DFLNH and LNnT with HAZ, FMI and weight velocity from 0–5 months [36]. These differences may be due to variations in individual HMO concentrations, for example the concentration of DFLNH is two-times higher in our study compared to others [36]; one study has not reported the concentrations [87]. Although differences exist between studies, specific HMOs such as DFLNH and LNnT consistently appear to be playing a protective role by regulating the accumulation of fat, thus potentially protecting infants from later-life obesity.

Foremost, in infants of non-secretor mothers, we observed negative associations between the concentration of FLNH and length and LAZ, and positive associations between DFLNT and infant weight, length, LAZ, WAZ and FM. These associations have not been reported previously [35]. Instead, negative associations have been observed between 6′SL and weight *z*-scores in infants aged 3–12 months born to non-secretor mothers [35], positive associations between DFLac, 3′SL and HAZ in infants born to secretor mothers [36], and positive associations between 3′SL and WAZ, irrespective of maternal secretor status [38], suggesting that the use of concentrations may produce misleading results. In our study, infants born to secretor mothers consuming higher concentrations of 3′SL had higher FFM. Sialylated HMOs such as 3′SL have been positively associated with infant growth (length, HAZ and WAZ) [36,38], although secretor status was not accounted for. While the relationship suggests links between HMOs and growth, it should be noted that the direct supplementation of the breastfed infant may have an additive effect that could have negative consequences by accelerating growth beyond the normal trajectory. This raises the potential for individualized supplementation in premature or sick infants or those failing to thrive.

Traditionally, growth comparisons are made between breastfed and formula-fed infants, with both showing different growth patterns. The growth patterns of breastfeeding infants are considered to be more conducive to optimal growth and development [88,89] and this may be due in part to the highly variable bacterial and HMO content of breast milk. The addition of HMOs to infant formula produces conflicting results. For example, infants that consume formula containing 2′FL and LNnT showed no differences in weight gain and growth, despite having a lower incidence of respiratory tract infections, compared with controls fed formula containing only galacto-oligosaccharides [90]. Similarly, infants consuming formula containing 2′FL only showed no growth differences compared to infants fed a control formula and breastfed infants. [91]. However, a recent study reported a positive relationship between 2′FL and 0–5 month weight velocity and FMI at five months [36]. Despite high concentrations of 2′FL in our study, we did not observe similar relationships suggesting that 2′FL alone may be associated with weight gain when used in formula compared to multiple HMOs. Taken together, the emerging evidence supports a role for HMOs in infant growth and development. Given that rapid and excessive weight gain is considered a risk factor for childhood and adult obesity, it is important to investigate the supplementation of HMOs in greater detail, particularly if supplementation is given to breastfed infants. Future studies, however, should take into account multiple human milk components when modelling the impact of human milk composition and volume on infant growth and body composition [92].

Human milk intake is related to infant growth and body composition development [39,93], however, studies that measure infant milk intake and intakes of human milk components are not common. We observed positive associations between the intake of individual fucosylated and sialylated HMOs and infant body composition. This is consistent with a recent cohort of exclusively breastfed infants (*n* = 140) which reported associations between intakes of 3FL, 3′SL, 6′SL, LNFP II, LNFP III, LSTb and DSLNH and infant FM, intakes of LNFP II and DSLNH and WAZ and intakes of 3FL, 3′SL, LNFP II, LSTb and DSLNH and WLZ [19], however they did not find relationships of DFLac, DFLNT, FDSLNH or 2′FL with weight, BMI, BMIAZ, FFM, FFMI and FM as we did. Conversely, we could not replicate their findings regarding relationships between HMO intakes and infant FM, WLZ, and WAZ. Of note is that the infants in the Saben et al. study [19] had lower 24 h milk intakes (643 ± 27 mL) compared to our study (785 ± 172 mL). Although associations differ between the current and previous study, it is more plausible that HMO intakes, rather than concentrations, would moderate the infant growth.

The findings of the current study need to be confirmed in larger longitudinal cohorts to understand how HMO intakes contribute to infant growth. Furthermore, it is well established that HMO profiles differ based on secretor status [44]. As expected, the directionality of associations was different between secretors and non-secretors. In infants of secretor mothers, 3′SL intake was positively associated with weight, length, FFM, FFMI and WAZ, while in infants of non-secretor mothers 6′SL and FDSLNH intakes were negatively associated with weight, FFM, adiposity, and *z*-scores. Although these data suggest a growth regulating role of sialylated HMOs, maternal secretor status appears to play a major role and the findings and needs to be confirmed in larger longitudinal cohorts by stratifying data according to secretor status when reporting on infant growth and development outcomes.

Finally, we have shown in our entire cohort that maternal adiposity was negatively associated with infant adiposity, while lower FFM and FM parameters were observed in infants of non-secretor mothers with higher FFM. Taken together, our results suggest that maternal body composition impacts infant body composition, and this relationship is mediated by HMO composition and intake, along with human milk’s microbial profile [94,95]. Unfortunately, this study did not identify the mediating factors, suggesting it may have been underpowered to do so. Much larger cohorts may be able to elucidate the biological pathways in more detail.

This study provides additional evidence that maternal body composition plays a significant role in influencing human milk microbiota, HMO composition and infant body composition. This presents a potential opportunity for modulating maternal body composition through diet [72,73], which may subsequently modulate human milk components composition and thereby impacting infant body composition. Furthermore, in addition to breastfeeding, the supplementation with HMOs [96] and probiotic bacteria [97,98] to compromised infants may promote optimal growth, body composition development and infant gut colonization.

The strengths of this study are that infants were exclusively breastfed during the first three months of life, negating the need to account for formula feeding, which is known to have a profound effect on the development of infant body composition [99,100]. Additionally, measurements of 24-h milk intake and daily HMO intake allowed for a deeper understanding of the relationships between HMO and infant growth compared to using HMO concentrations only. Furthermore, our study used the same HPLC analytical platform as most of the previous studies, allowing the ability to compare results between studies unencumbered by the potential bias caused by differences in analytical platforms. Furthermore, we have measured maternal body composition in addition to BMI. However, our study does have some limitations. The number of participating dyads in this pilot study was relatively small, and stratification of data based on maternal secretor status left only 11 participants in the non-secretor group, therefore results should be interpreted cautiously and be considered as a framework for future studies. Also, the cross-sectional nature of the study meant that we were unable to speculate if the observed relationships would be maintained over time. Furthermore, whilst bioimpedance spectroscopy is an improved method of body composition measurement compared to anthropometrics and within a population, we were unable to access more resource heavy reference methods for this study. Our population consisted of term, healthy, exclusively breastfed infants from predominantly Caucasian mothers of high social-economic status living in Western Australia; therefore, the results may not be transferable to other populations.

## 4. Materials and Methods

### 4.1. Study Design

Pregnant women were recruited during the third trimester of pregnancy (>30 weeks’ gestation) as part of the BLOSOM (Breastfeeding Longitudinal Observational Study of Mothers and kids) cohort study. Study design, participant characteristics, and data collection have been described previously [48]. Briefly, this involved healthy (self-reported) women (*n* = 60) with no major pregnancy complications, exclusively breastfeeding at the time of sample collection. Exclusion criteria were infant factors that could potentially influence growth and development of body composition, maternal smoking, and pregnancy complications such as preterm labor, preeclampsia, and gestational diabetes mellitus. All mothers provided informed written consent to participate in the study, which was approved by the Human Research Ethics Committee at The University of Western Australia (RA/4/20/4023).

### 4.2. Sample Collection

Human milk collection has been described previously [48]. Briefly, this involved mothers washing their hands thoroughly with soap and water and cleaning the nipple and areola of the expressing breast with alcohol and chlorhexidine prep pads (70% isopropyl alcohol and 2% chlorhexidine digluconate, Reynard Health Supplies, Artarmon, NSW, Australia), followed by rinsing with sterile saline solution (Livingstone, Mascot, NSW, Australia) and drying with sterile gauze swabs (Livingstone, Mascot, NSW, Australia). 10–20 mL of human milk was hand-expressed directly into sterile tubes (Greiner Bio-One, Kremsmünster, Austria). Human milk samples were stored at 4 °C in the fridge at the participant’s home before being collected within 24 h and transported on ice to the laboratory, where they were immediately aliquoted into sterile tubes (Sarstedt, Numbrecht, Germany) and stored at −80 °C until further analysis.

### 4.3. Human Milk Oligosaccharides Analysis

100 μL human milk aliquots from each participant were sent to the Bode Lab at the University of California San Diego, (San Diego, CA, USA), on dry ice. The concentration and composition of HMOs in human milk samples was analyzed by high-performance liquid chromatography (HPLC) after labelling with the fluorescent tag 2-aminobenzamide as described previously [80]. The following 19 HMOs were identified and quantified: 2′FL, 3FL, 3′SL, 6′SL, DFLac, DFLNH, DFLNT, DSLNH, DSLNT, FDSLNH, FLNH, LNFP I, LNFP II, LNFP III, LNH, LNnT, LNT, LSTb, and LSTc. Maternal secretor status was identified based on the presence or near-absence of 2′FL or LNFP I in human milk [5].

### 4.4. 24-h Milk Intake

Infant 24-h milk intake was measured at 3 months’ postpartum (mean ± SD: 3.3 ± 0.6 months; range: 1.1–5.0) by mothers in their homes using the 24-h test-weighing protocol as described previously [101]. Three-month 24-h milk intakes were considered representative of intakes during the exclusive breastfeeding period, as there is no significant variation in human milk intake from one to six months within infants [102].

### 4.5. Calculated Daily Intakes of Human Milk Components

Calculated daily intakes of HMOs (µg) were determined as the concentration of the HMOs (µg/mL) multiplied by 24-h milk intake that was converted from g to mL using a density of human milk of 1.03 g/mL [103].

### 4.6. Anthropometry and Body Composition Measurements

Anthropometric and body composition measurements were performed for both mothers and infants at three months’ postpartum (mean ± SD: 3.1 ± 0.1 months; range: 2.9–3.5) and have been described previously [48]. Maternal and infant body composition was measured with bioelectrical impedance spectroscopy using a Impedimed SFB7 battery-operated bioelectrical impedance analyser (ImpediMed, Brisbane, Australia) according to the protocols described previously by Gridneva et al. [94,104].

Briefly, this involved wiping the dorsal surface of hand and foot with isopropyl alcohol before applying the single use Ag–AgCl gel proximal electrodes (ImpediMed) on the skin. For mothers, the electrodes were placed 5 cm apart at the metacarpophalangeal joints of the hand and ulnar styloid process on the wrist, and at the lateral malleolus of the ankle and metatarsal-phalangeal joints on the foot. A series of 10 consecutive measurements were taken within 1–2 min in supine position on a non-conductive surface.

Infant body composition was using the same protocol as for mothers except that the electrodes were placed 3 cm apart. Infants were wearing a dry diaper and a singlet at the time of measurement and insulating material (towel) was used to prevent skin-to-skin contact between infant’s limbs and torso. A series of 10–50 consecutive measurements were taken within 1–3 min with infants in supine position on a non-conductive surface. Resistance (ohm) at 50 kHz (R_50_) was determined from the curve of best fit, averaged for analysis purposes and used in the Lingwood et al. [40] bioelectrical impedance spectroscopy equations for FFM.

In addition to standard body composition measurements (FFM, FM, and %FM), the height-normalized body composition indices of mothers and infants (FFMI, FMI) were calculated [105] as well as FM to FFM ratio (FM/FFM). Infant *z*-scores (WLZ, WAZ, LAZ, BMIAZ and HCAZ) were determined using the World Health Organisation (WHO) Anthro software v3.2.2 [106].

### 4.7. DNA Extraction and Quantitation

Bacterial DNA was extracted from 1 mL human milk samples using the QIAGEN MagAttract Microbial DNA Isolation Kit (Qiagen, Chadstone, Australia) on the Kingfisher Flex platform following the manufacturer’s instructions, as described previously [107]. Total DNA yield was assessed using the Qubit^®^ dsDNA High Sensitivity Assay (Invitrogen, Mulgrave, VIC, Australia) on a Qubit^®^ 2.0 Fluorometer (Life Technologies, Mulgrave, VIC, Australia) according to manufacturer’s instructions. The limit of detection was 10 pg/μL.

### 4.8. 16S rRNA Gene Amplification and Barcoding

The full-length 16S rRNA gene was amplified using the primer pair 27F and 1492R with a universal UNITAG sequence and amine block attached to the 5′ ends of each primer as previously reported [108,109].

The primary PCR was carried out in 25 µL reactions containing 5 µL template or nuclease-free water (negative template control), 1X AccuStart II ToughMix (Quantabio, Beverly, MA, USA), 0.625 µL each of dsDNase and DTT (ArcticZymes PCR decontamination kit, Tromsø, Norway) and 0.3 μM each of the forward and reverse primers. An ArcticZymes PCR decontamination kit was used to remove contaminating DNA in PCR master mixes without reduction of PCR sensitivity; activation and inactivation of ArcticZymes dsDNase was performed as described previously [109]. The PCR amplification conditions consisted of an initial heating step at 94 °C for 3 min; 35 cycles of 94 °C for 30 s, 52 °C for 30 s, and 72 °C for 2 min; and a final extension step of 72 °C for 5 min. Primary PCR products were quantified on a Qubit^®^ 2.0 Fluorometer and visualized on a QIAxcel capillary gel electrophoresis system using a DNA high-resolution gel cartridge (run parameters OM500) to confirm the presence and size of amplicons. Primary PCR products were purified using NucleoMag NGS magnetic beads (Macherey-Nagel, Düren, Germany), normalized to 1 ng/µL, and used as template for the barcoding PCR.

Primary PCR products were barcoded using UNITAG barcoded 1F-5F and 16R-30R primers. PCR was carried out in 20 µL reactions containing 2 µL of template or nuclease-free water (negative template control), 1X AccuStart II ToughMix and 0.3 μM each of the forward and reverse barcoded primers. PCR cycling conditions were the same as described above, but with 10 cycles.

Barcoded PCR amplicons were pooled in equimolar concentrations based on Qubit quantitation. The amplicon pool was loaded onto a 1.2% agarose gel using SYBR Safe DNA stain (Invitrogen) to separate the targeted ~1500 bp band. Amplicon pools were gel purified using a QIAquick gel extraction kit (QIAGEN) according to the manufacturer’s protocol. ~500 ng of DNA (pooled amplicons) was used for library preparation.

### 4.9. PacBio Sequencing

Purified amplicon pools were sequenced at the Australian Genome Research Facility (AGRF) at The University of Queensland (UQ), QLD, Australia. SMRTbell adapters were ligated onto barcoded PCR products and the libraries were sequenced using Pacific Biosciences single molecule real-time (SMRT) hi-fidelity (HiFi) sequencing on a single SMRT cell using the PacBio Sequel II System. Raw data were processed at AGRF-UQ using PacBio SMRTLink to generate demultiplexed .fastq files.

### 4.10. Sequencing Data Processing

Full-length 16S rRNA gene sequence data was processed using mothur v.1.44.3 [110] (as previously described [107]) on the Kaya supercomputer (Pawsey Supercomputing Centre). Briefly, .fastq files were converted and merged to a single .fasta file. The merged .fasta file was length filtered (1336–1743 bp) and sequences containing homopolymers of >9 bases were removed. Sequences were aligned to the SILVA reference alignment v138. Sequences were classified using classify.seqs with the SILVA taxonomy database v138 and a confidence threshold of 80. Based on classification, non-bacterial sequences were filtered and discarded from the dataset. OTUs were created using the cluster.split command with a 0.03 similarity cut-off value. Subsampling was performed at 403 reads based on an average Good’s coverage value of 96.5%. Reads from negative extraction controls and negative PCR controls are provided in Table S7. The representative sequences of the most abundant OTUs in human milk were analysed to identify bacterial species using the Basic Local Alignment Search Tool (BLAST) program and associated NCBI database [111]. The cut-off for alignment percent identity was set at >98% and all sequencing reads percent identity was between 98.30–100%.

### 4.11. Statistical Analysis

Data were analysed using the R environment for statistical computing [112]. Categorical variables are summarised as counts and percentages, and normally distributed continuous variables are summarised with means and standard deviations (SD), while non-normally distributed variables are summarised with medians and inter-quartile ranges (IQR). HMO concentrations and intake variables, as well as infant and maternal body composition variables, were assessed for normality both visually and with the Shapiro Wilk test for normality, and skewed variables were log-transformed prior to analysis. Welch’s *t*-tests for unequal variances were performed to determine differences in continuous maternal and infant characteristics, and HMO concentrations and intakes between non-secretor and secretor mothers. Additionally, Fisher’s exact tests were used to compare parity (0, 1, 2+) and delivery mode between secretor status.

An additional seven groups of analyses were performed to assess relationships between (a) maternal body composition measurements and concentrations of HMOs, (b) maternal body composition measurements and human milk OTU relative abundances, (c) maternal and infant body composition measurements, (d) concentrations of HMOs and human milk OTU relative abundances, (e) human milk OTU relative abundances and infant body composition measurements, (f) concentrations of HMOs and infant body composition measurements, and (g) intakes of HMOs and infant body composition measurements. To aid presentation of the model coefficients, the concentrations and intakes of 2′FL and DFLNT and the intake of 3FL were divided by 100 prior to analyses, while the intake and concentration of 6′SL and FLNH and the intake of 3′SL were divided by 10 prior to analyses. Bacterial OTUs belonging to the category “others” were not included in statistical analysis due to their collective percentage forming <1% and the large number of OTUs comprising this category. Predominant OTUs represent any OTU forming >1% relative abundance in a sample.

For the three analyses considering HM OTU relative abundances (analyses (b), (d) and (e)), generalised additive models, for location, scale and shape were fitted. This type of modelling is a framework for fitting regression-type models that allows the response variables, in this case the relative abundances, to follow any distribution. We allowed the relative abundances to follow a zero-inflated beta distribution, as this has been shown to be appropriate for this type of data [113]. OTUs which made up ≥ 1% of the total relative abundance in the samples and satisfied a prevalence threshold of presence in at least ≥10% of samples were considered for these analyses.

For the other four analyses which did not consider HM OTU relative abundances (analyses (a), (c), (f) and (g)), general linear regression models with separate variances for secretors and non-secretors were fit for all continuous variables. When considering the two categorical variables of parity and mode of delivery, an ANOVA was fit.

For all seven analyses (a–g), an initial model was fit which included the predictor of interest, secretor status and an interaction between these two variables. The interaction was included to determine whether the relationships between the predictor and response variables varied between secretor statuses. Additionally, model (e), which looked at the relationship between OTUs and body composition, also included a variable representing the volume of milk intake. Model selection was conducted using the Akaike information criterion (AIC), and results from the final model are presented. Specifically, the estimated variable coefficients, standard errors, and *p*-values are provided. Due to the investigative nature of this cross-sectional study, no power calculation or *p*-value adjustment for multiple comparisons were performed.

## 5. Conclusions

The findings from this investigative study consolidate the relationships between HMOs and infant growth, suggesting that HMO intakes may have a significant influence on infant growth and body composition development during the first three months of life, highlighting the importance of daily intakes, and that these relationships differ based on maternal secretor status. Furthermore, maternal body composition influences specific human milk microbiota and HMOs, which may have implications for infant body composition development.

## Figures and Tables

**Table 1:** Associations between maternal body composition and HMO concentrations.

Maternal Predictor	Secretor			Non-Secretor			Interaction
	Parameter Estimate	SE	*p*-Value	Parameter Estimate	SE	*p*-Value	*p*-Value
	2′FL						
FM (%)	0.43	0.16	**0.01**	0.07	0.03	**0.01**	0.03
Log (FMI (kg/m^2^))	6.05	3.00	0.05	1.25	0.53	**0.02**	0.23
Log (FM/FFM)	7.85	4.11	0.06	1.59	0.61	**0.01**	0.14
	3′SL						
Log (FM (kg))	−0.34	1.48	0.82	−7.13	3.03	**0.02**	0.05
FM (%)	−0.04	0.08	0.66	−0.37	0.12	**<0.001**	0.02
Log (FMI (kg/m^2^))	−0.63	1.53	0.68	−5.88	2.56	**0.03**	0.08
Log (FM/FFM)	−2.81	2.04	0.17	−8.36	2.68	**<0.001**	0.11
	FLNH						
Age	−0.12	0.24	0.63	1.80	0.69	**0.01**	0.01
Log (Weight (kg))	14.32	5.43	**0.01**	−36.11	22.83	0.12	0.04
Log (BMI (kg/m^2^))	13.81	5.99	**0.03**	−15.11	15.95	0.35	0.10
Log (FM (kg))	8.37	3.35	**0.02**	−16.06	8.88	0.08	0.01
Log (FMI (kg/m^2^))	7.86	3.50	**0.03**	−11.22	7.82	0.16	0.03
	Entire cohort						
	2′FL						
Log (Weight (kg))	3.63	1.52	**0.02**	-	-	-	-
Log (BMI (kg/m^2^))	2.37	1.04	**0.03**	-	-	-	-
Log (FM (kg))	1.68	0.61	**0.01**	-	-	-	-
	Log (LNH)						
Log (BMI (kg/m^2^))	0.73	0.27	**0.01**	-	-	-	-
FFMI (kg/m^2^)	0.05	0.02	**0.02**	-	-	-	-

Data are parameter value ± standard error of measurement (SE). When the interaction term with secretor status was present in the final model, results were broken down by secretor and non-secretor groups, whereas when the interaction term was not present in the final model, results are presented for the entire cohort. Significant *p*-values are indicated by bold text. 2′FL—2′-fucosyllactose; 3′SL—3′-sialyllactose; BMI—body mass index; FFMI—fat-free mass index; FLNH—fucosyllacto-N-hexaose; FM—fat mass; FM/FFM—fat mass to fat-free mass ratio; FMI—fat mass index; LNH—lacto-N-hexaose.

**Table 2:** Associations between maternal body composition and human milk bacterial OTU relative abundance.

Maternal Predictor	Secretor			Non-Secretor			Interaction
	Parameter Estimate	SE	*p*-Value	Parameter Estimate	SE	*p*-Value	*p*-Value
	OTU03 (*Streptococcus salivarius*)						
Log (Weight (kg))	−2.24	0.82	**0.01**	6.01	5.07	0.24	0.12
Log (BMI (kg/m^2^))	−2.52	0.89	**0.01**	4.44	2.8	0.12	0.02
Log (FM (kg))	−1.37	0.5	**0.01**	2.63	2	0.19	0.06
FM (%)	−0.06	0.03	**0.04**	0.12	0.08	0.17	0.05
Log (FMI (kg/m^2^))	−1.42	0.52	**0.01**	2.25	1.51	0.14	0.02
Log (FM/FFM)	−1.33	0.64	**0.04**	2.66	1.96	0.18	0.06
	OTU06 (*Streptococcus lactarius*)						
Age	0.03	0.04	0.41	0.82	0.26	**<0.001**	<0.001
	OTU08 (*Gemella haemolysans*)						
Log (BMI (kg/m^2^))	−0.69	1.28	0.59	5.43	2.39	**0.03**	0.03
FFMI (kg/m^2^)	−0.05	0.11	0.64	0.55	0.24	**0.03**	0.03
Log (FMI (kg/m^2^))	−0.37	0.74	0.62	2.62	1.22	**0.04**	0.04
	OTU10 (*Veillonella nakazawae*)						
FFMI (kg/m^2^)	0.12	0.06	0.04	0.42	0.19	**0.03**	0.14
	Entire cohort						
	OUT01 (*Streptococcus mitis*)						
Age	0.07	0.03	**0.04**	-	-	-	-
	OTU02 (*Staphylococcus epidermidis*)						
Log (Weight (kg))	−1.54	0.69	**0.03**	-	-	-	-
FFM (kg)	−0.04	0.02	**0.02**	-	-	-	-
Log (FM (kg))	−1.04	0.43	**0.02**	-	-	-	-
Log (FMI (kg/m^2^))	−0.9	0.43	**0.04**	-	-	-	-
	OTU10 (*Veillonella nakazawae*)						
FFM (kg)	0.04	0.02	**0.03**	-	-	-	-

Data are parameter value ± standard error of measurement (SE); note that these estimates are on the logit scale. When the interaction term with secretor status was present in the final model, results were separated by secretor and non-secretor groups, whereas when the interaction term was not present in the final model, results were presented for the entire cohort. Significant *p*-values are indicated by the bold text. BMI—body mass index; ECS—emergency caesarean section; FFM—fat-free mass; FFMI—fat-free mass index; FM—fat mass; FM/FFM—fat mass to fat-free mass ratio; FMI—fat mass index; MOD—mode of delivery.

**Table 3:** Associations between maternal and infant body composition at three months postpartum.

Maternal Predictors	Secretor			Non-Secretor			Interaction
	Parameter Value	SE	*p*-Value	Parameter Value	SE	*p*-Value	*p*-Value
	Infant weight (kg)						
FFM (kg)	0.01	0.01	0.51	−0.26	0.12	**0.04**	0.03
FFMI (kg/m^2^)	−0.00	0.04	0.98	−0.47	0.14	**<0.001**	<0.001
	Infant length (cm)						
FFM (kg)	0.10	0.04	**0.01**	−0.66	0.36	0.08	0.04
FFMI (kg/m^2^)	0.19	0.13	0.16	−1.04	0.47	**0.03**	0.02
	Infant BMI (kg/m^2^)						
FFMI (kg/m^2^)	−0.11	0.07	0.11	−0.63	0.2	**<0.001**	0.01
	Infant log (FFM (kg))						
FFM (kg)	0.00	0.00	0.33	−0.04	0.02	**0.03**	0.02
FFMI (kg/m^2^)	0.00	0.01	0.83	−0.06	0.02	**<0.001**	<0.001
	Infant log (FFMI (kg/m^2^))						
Log (BMI (kg/m^2^))	−0.08	0.07	0.23	−0.34	0.15	**0.03**	0.13
FFM (kg)	0.00	0.00	0.97	−0.02	0.01	**0.03**	0.03
FFMI (kg/m^2^)	−0.00	0.01	0.70	−0.05	0.01	**<0.001**	<0.001
	Infant FM (kg)						
FFMI (kg/m^2^)	0.01	0.02	0.44	−0.16	0.06	**0.01**	0.01
	Infant FMI (kg/m^2^)						
FFMI (kg/m^2^)	0.03	0.05	0.60	−0.42	0.16	**0.01**	0.01
	Entire cohort						
	Infant length (cm)						
Log (Weight (kg))	3.48	1.69	**0.04**	-	-	-	-
	Infant BMI (kg/m^2^)						
Log (BMI (kg/m^2^))	−2.16	0.9	**0.02**	-	-	-	-
Log (FM (kg))	−1.24	0.51	**0.02**	-	-	-	-
FM (%)	−0.06	0.03	**0.03**	-	-	-	-
Log (FMI (kg/m^2^))	−1.33	0.51	**0.01**	-	-	-	-
	Infant FM (%)						
Age	−0.17	0.07	**0.02**	-	-	-	-
	Infant FM/FFM						
Age	−0.00	0.00	**0.02**	-	-	-	-

Data are parameter value ± standard error of measurement (SE). When the interaction term with secretor status was present in the final model, results are separated by secretor and non-secretor groups, whereas when the interaction term was not present in the final model, results are presented for the entire cohort. Significant *p*-values are indicated by the bold text. BMI—body mass index; FFM—fat-free mass; FFMI—fat-free mass index; FM—fat mass; FM/FFM—fat mass to fat-free mass ratio; FMI—fat mass index.

**Table 4:** Associations between individual HMO concentrations and bacterial OTUs at two months postpartum.

HMO (Concentrations)	Secretor			Non-Secretor			Interaction
	Parameter Estimate	SE	*p*-Value	Parameter Estimate	SE	*p*-Value	*p*-Value
	OTU02 (*Staphylococcus epidermidis*)						
3′SL	−0.12	0.05	**0.01**	0.09	0.11	0.45	0.10
	OTU04 (*Cutibacterium acnes*)						
6′SL	−0.01	0.02	0.71	0.01	0.05	**0.03**	0.04
	OTU05 (*Streptococcus parasanguis*)						
Log (DFLac)	0.03	0.37	0.93	3.09	1.17	**0.01**	0.02
3′SL	0.05	0.05	0.34	−2.80	0.78	**<0.001**	<0.001
6′SL	−0.02	0.05	0.60	−0.20	0.09	**0.04**	0.10
Log (LNFP I)	−0.11	0.31	0.72	−9.49	2.40	**<0.001**	<0.001
Log (LSTb)	0.05	0.41	0.90	−17.23	7.23	**0.02**	0.02
Log (LSTc)	0.11	0.55	0.84	−1.71	0.81	**0.04**	0.07
Log (LNH)	−0.04	0.36	0.91	−4.32	1.37	**<0.001**	<0.001
DSLNH	0.00	0.01	0.73	−0.02	0.01	**0.04**	0.05
	OTU06 (*Streptococcus lactarius*)						
2′FL	0.02	0.04	0.65	26.81	11.68	**0.03**	0.03
Log (LNnT)	0.28	0.55	0.61	−1.89	0.58	**<0.001**	0.01
Log (FDSLNH)	−0.03	0.18	0.85	−2.15	0.69	**<0.001**	<0.001
	OTU08 (*Gemella haemolysans*)						
2′FL	−0.01	0.04	0.83	0.98	0.33	**<0.001**	<0.001
3′SL	0.06	0.09	0.51	−0.25	0.11	**0.03**	0.04
Log (LNnT)	−0.19	0.39	0.63	1.22	0.54	**0.03**	0.04
	OTU10 (*Veillonella nakazawae*)						
Log (DFLac)	−1.81	0.85	**0.04**	−0.33	0.35	0.35	0.11
	Entire cohort						
	OTU02 (*Staphylococcus epidermidis*)						
FLNH	−0.04	0.02	**0.03**	-	-	-	-
	OTU03 (*Streptococcus salivarius*)						
FLNH	−0.04	0.02	**0.02**	-	-	-	-
	OTU04 (*Cutibacterium acnes*)						
Log (LNnT)	0.71	0.28	**0.01**	-	-	-	-
	OTU08 (*Gemella haemolysans*)						
Log (DFLac)	0.47	0.19	**0.02**	-	-	-	-
	OTU10 (*Veillonella nakazawae*)						
Log (LNFP III)	−0.83	0.36	**0.03**	-	-	-	-
DSLNT	−0.01	0.00	**<0.001**	-	-	-	-
Log (FDSLNH)	−0.43	0.17	**0.02**	-	-	-	-

Data are parameter estimate ± standard error of measurement (SE), note that these estimates are on the logit scale. When the interaction term with secretor status was present in the final model, results were separated by secretor and non-secretor groups, whereas when the interaction term was not present in the final model, results were presented for the entire cohort. Significant *p*-values are indicated by the bold text. 2′FL—2′-fucosyllactose; 3′SL—3′-sialyllactose; 6′SL—6′-sialyllactose; DFLac—difucosyllactose; DSLNH—disialyllacto-N-hexaose; DSLNT—disialyllacto-N-tetraose; FDSLNH—fucodisialyllacto-N-hexaose; FLNH—fucosyllacto-N-hexaose; LNFP I—lacto-N-fucopentaose I; LNFP III—lacto-N-fucopentaose III; LNH—lacto-N-hexaose; LNnT—lacto-N-neotetraose; LSTb—sialyl-lacto-N-tetraose b; LSTc—sialyl-lacto-N-tetraose c; NA—not available.

**Table 5:** Associations between bacterial OTUs and infant body composition at three months postpartum.

Human Milk OTUs	Secretor			Non-Secretor			Interaction	Log (Infant Milk Intake)
	Parameter Value	SE	*p*-Value	Parameter Value	SE	*p*-Value	*p*-Value	*p*-Value
	Head circumference (cm)							
OTU01 (*Streptococcus mitis*)	0.01	0.01	0.56	−0.03	0.01	**0.01**	0.03	0.03
	Length-for-age *z*-score							
OTU01 (*Streptococcus mitis*)	−0.00	0.01	0.61	−0.02	0.01	**0.03**	0.15	0.03
	BMI-for-age *z*-score							
OTU05 (*Streptococcus parasanguis*)	−0.02	0.02	0.42	0.01	0.01	**0.02**	0.16	0.03
	Head circumference-for-age *z*-score							
OTU01 (*Streptococcus mitis*)	0.00	0.01	0.63	−0.03	0.01	**<0.001**	0.01	0.10
	Entire cohort							
	Weight (kg)							
OTU05 (*Streptococcus parasanguis*)	0.02	0.01	**<0.001**	-	-	-	-	<0.001
	Length (cm)							
OTU02 (*Staphylococcus epidermidis*)	−0.04	0.02	**0.04**	-	-	-	-	<0.001
	Weight-for-length *z*-score							
OTU02 (*Staphylococcus epidermidis*)	0.02	0.01	**0.02**	-	-	-	-	NA
OTU06 (*Streptococcus lactarius*)	0.02	0.01	**0.04**	-	-	-	-	NA
	Weight-for-age *z*-score							
OTU05 (*Streptococcus parasanguis*)	0.01	0.01	**0.03**	-	-	-	-	<0.001
	BMI-for-age *z*-score							
OTU06 (*Streptococcus lactarius*)	0.02	0.01	**0.01**	-	-	-	-	NA
	Log fat-free mass (kg)							
OTU05 (*Streptococcus parasanguis*)	0.00	0.00	**<0.001**	-	-	-	-	<0.001
	Log fat-free mass index (kg/m^2^)							
OTU05 (*Streptococcus parasanguis*)	0.00	0.00	**0.01**	-	-	-	-	<0.001
	Fat mass (kg)							
OTU05 (*Streptococcus parasanguis*)	0.01	0.00	**0.03**	-	-	-	-	<0.001
OTU06 (*Streptococcus lactarius*)	0.01	0.00	**0.03**	-	-	-	-	0.01
	Fat mass index (kg/m^2^)							
OTU05 (*Streptococcus parasanguis*)	0.01	0.01	**0.03**	-	-	-	-	<0.001
OTU06 (*Streptococcus lactarius*)	0.02	0.01	**0.02**	-	-	-	-	0.03
	Fat mass (%)							
OTU06 (*Streptococcus lactarius*)	0.05	0.03	**0.04**	-	-	-	-	NA
	Fat mass to fat-free mass ratio							
OTU06 (*Streptococcus lactarius*)	0.00	0.00	**0.03**	-	-	-	-	NA

Data are parameter estimate ± standard error of measurement (SE); note that these estimates are on the logit scale. When the interaction term with secretor status was present in the final model, results were separated by secretor and non-secretor groups, whereas when the interaction term was not present in the final model, results were presented for the entire cohort. Significant *p*-values are indicated by the bold text.

**Table 6:** Associations between HMO concentrations and infant body composition at three months postpartum.

HMO Concentration	Secretor			Non-Secretor			Interaction
	Parameter Value	SE	*p*-Value	Parameter Value	SE	*p*-Value	*p*-Value
	Weight (kg)						
DFLNT	0.03	0.03	0.42	0.30	0.14	**0.04**	0.06
	Length (cm)						
DFLNT	−0.03	0.11	0.80	0.95	0.38	**0.01**	0.02
FLNH	−0.02	0.05	0.67	−0.22	0.09	**0.02**	0.06
	Weight-for-age *z*-score						
DFLNT	0.03	0.04	0.39	0.33	0.16	**0.04**	0.07
	Length-for-age *z*-score						
DFLNT	−0.01	0.05	0.82	0.43	0.19	**0.03**	0.03
FLNH	−0.01	0.02	0.76	−0.09	0.04	**0.04**	0.09
	Log fat-free mass (kg)						
3′SL	0.01	0.01	**0.04**	−0.01	0.01	0.41	0.13
	Fat mass (kg)						
DFLNT	0.01	0.01	0.58	0.12	0.06	**0.04**	0.06
	Entire cohort						
	Weight (kg)						
FLNH	−0.03	0.01	**0.04**	-	-	-	-
Log (DFLNH)	0.24	0.11	**0.03**	-	-	-	-
	Length (cm)						
Log (LNnT)	−1.11	0.5	**0.03**	-	-	-	-
Log (DFLNH)	1.1	0.36	**<0.001**	-	-	-	-
	Length-for-age *z*-score						
Log (LNnT)	−0.51	0.23	**0.03**	-	-	-	-
Log (DFLNH)	0.44	0.17	**0.01**	-	-	-	-
	Log fat-free mass (kg)						
Log (DFLNH)	0.04	0.02	**0.02**	-	-	-	-
	Fat mass (%)						
Log (LNFP III)	−1.6	0.64	**0.02**	-	-	-	-
	Fat mass to fat-free mass ratio						
Log (LNFP III)	−0.03	0.01	**0.02**	-	-	-	-

Data are parameter value ± standard error of measurement (SE). When the interaction term with secretor status was present in the final model, results were separated by secretor and non-secretor groups, whereas when the interaction term was not present in the final model, results were presented for the entire cohort. Significant *p*-values are indicated by the bold text. 3′SL—3′-sialyllactose; DFLNH—difucosyllacto-N-hexaose; DFLNT—difucosyllacto-N-tetrose; FLNH—fucosyllacto-N-hexaose; LNFP III—lacto-N-fucopentaose III; LNnT—lacto-N-neotetraose.

**Table 7:** Associations between individual HMO intake and infant body composition at three months postpartum.

HMOs (Intake)	Secretor			Non-Secretor			Interaction
	Parameter Value	SE	*p*-Value	Parameter Value	SE	*p*-Value	*p*-Value
	Weight (kg)						
Log (3′SL)	0.88	0.28	**<0.001**	−0.31	0.36	0.39	0.01
6′SL	−0.01	0.03	0.86	−0.09	0.04	**0.03**	0.11
	Length (cm)						
Log (3′SL)	2.59	1.01	**0.01**	−0.58	1.16	0.62	0.05
	Body mass index (kg/m^2^)						
Log (FDSLNH)	−0.17	0.21	0.44	−2.87	1.06	**0.01**	0.02
	Weight-for-length *z*-score						
Log (FDSLNH)	−0.22	0.14	0.13	−1.54	0.61	**0.02**	0.04
	Weight-for-age *z*-score						
Log (3′SL)	0.86	0.35	**0.02**	−0.20	0.39	0.62	0.05
6′SL	0.01	0.04	0.70	−0.11	0.04	**0.01**	0.03
	BMI-for-age *z*-score						
Log (FDSLNH)	−0.09	0.14	0.52	−1.50	0.56	**0.01**	0.02
	Log fat-free mass (kg)						
Log (3′SL)	0.14	0.04	**<0.001**	−0.05	0.05	0.34	0.01
	Log fat-free mass index (kg/m^2^)						
Log (3′SL)	0.09	0.03	**0.01**	−0.04	0.04	0.32	0.01
Log (FDSLNH)	(−)0.00	0.01	0.09	−0.19	0.07	**0.01**	0.02
	Fat mass (kg)						
6′SL	0.00	0.01	0.83	−0.04	0.02	**0.03**	0.07
	Fat mass index (kg/m^2^)						
6′SL	0.00	0.04	0.90	−0.01	0.00	**0.04**	0.09
	Entire cohort						
	Weight (kg)						
2′FL	0.00	0.00	**0.03**	-	-	-	-
3FL	0.09	0.03	**0.01**	-	-	-	-
Log (DFLac)	0.33	0.14	**0.02**	-	-	-	-
Log (DFLNH)	0.28	0.11	**0.01**	-	-	-	-
	Length (cm)						
3FL	0.20	0.08	**0.01**	-	-	-	-
Log (DFLNH)	1.22	0.35	**<0.001**	-	-	-	-
	Body mass index (kg/m^2^)						
Log (DFLac)	0.51	0.23	**0.03**	-	-	-	-
Log (LSTb)	0.83	0.39	**0.04**	-	-	-	-
DFLNT	0.12	0.05	**0.04**	-	-	-	-
	Weight-for-age *z*-score						
3FL	0.10	0.04	**0.01**	-	-	-	-
Log (DFLNH)	0.28	0.13	**0.03**	-	-	-	-
	Length-for-age *z*-score						
3FL	0.08	0.04	**0.04**	-	-	-	-
Log (DFLNH)	0.48	0.17	**0.01**	-	-	-	-
	BMI-for-age *z*-score						
Log (DFLac)	0.28	0.13	**0.04**	-	-	-	-
DFLNT	0.07	0.03	**0.03**	-	-	-	-
	Log fat-free mass (kg)						
3FL	0.01	0.00	**0.01**	-	-	-	-
Log (DFLac)	0.05	0.02	**0.02**	-	-	-	-
Log (DFLNH)	0.04	0.02	**0.01**	-	-	-	-
	Log fat-free mass index (kg/m^2^)						
3FL	0.01	0.00	**0.04**	-	-	-	-
Log (DFLac)	0.04	0.01	**0.02**	-	-	-	-
DFLNT	0.01	0.00	**0.03**	-	-	-	-
	Fat mass (kg)						
2′FL	0.02	0.01	**0.04**	-	-	-	-

Data are parameter value ± standard error of measurement (SE). When the interaction term with secretor status was present in the final model, results were separated by secretor and non-secretor groups, whereas when the interaction term was not present in the final model, results were presented for the entire cohort. Significant *p*-values are indicated by the bold text. 2′FL—2′-fucosyllactose; 3′SL—3′-sialyllactose; 3FL—3-fucosyllactose; 6′SL—6′-sialyllactose; DFLac—difucosyllactose; DFLNH—difucosyllacto-N-hexaose; DFLNT—difucosyllacto-N-tetrose; FDSLNH—fucodisialyllacto-N-hexaose; LSTb—sialyl-lacto-N-tetraose b.

## Data Availability

The data presented in this study are available from the corresponding author upon reasonable request.

## Supplementary Materials

The supporting information can be downloaded at: https://www.mdpi.com/article/10.3390/ijms23052865/s1.
