# Supplementary material for: Human Milk Oligosaccharides and Bacterial Profile Modulate Infant Body Composition during Exclusive Breastfeeding
Source: Int J Mol Sci. 2022 Mar 5;23(5):2865. doi: 10.3390/ijms23052865 (PMC8911220; doi:10.3390/ijms23052865)
Supplement: Supplementary file 1 [file ijms-23-02865-s001.zip › ijms-1539920-supplement.pdf]

International Journal of *Molecular Sciences*

MDPI

Article

Human Milk Oligosaccharides and Bacterial Profile Modulate Infant Body Composition during Exclusive Breastfeeding

Ali S. Cheema [1], Zoya Gridneva [1], Annalee J. Furst [2,3], Ana S. Roman [2,3], Michelle L. Trevenen [4], Berwin A. Turlach [4], Ching T. Lai [1], Lisa F. Stinson [1], Lars Bode [2,3], Matthew S. Payne [5,6] and Donna T. Geddes [1,*]

1 School of Molecular Sciences, The University of Western Australia, Crawley, WA 6009, Australia; alisadiq.cheema@research.uwa.edu.au (A.S.C.); zoya.gridneva@uwa.edu.au (Z.G.); ching-tat.lai@uwa.edu.au (C.T.L.); lisa.stinson@uwa.edu.au (L.F.S.);
2 Larsson-Rosenquist Foundation Mother-Milk-Infant Center of Research Excellence, University of California San Diego, La Jolla, CA 92093, USA; aloeffler@health.ucsd.edu (A.J.F.); acroman@sdsu.edu (A.S.R.); lbode@health.ucsd.edu (L.B.)
3 Department of Pediatrics, University of California San Diego, La Jolla, CA 92093, USA
4 Centre for Applied Statistics, The University of Western Australia, Crawley, WA 6009, Australia; michelle.trevenen@uwa.edu.au (M.L.T.); berwin.turlach@uwa.edu.au (B.A.T.)
5 Division of Obstetrics and Gynaecology, School of Medicine, The University of Western Australia, Subiaco, WA 6008, Australia; matthew.payne@uwa.edu.au
6 Women and Infants Research Foundation, Subiaco, WA 6008, Australia
* Correspondence: donna.geddes@uwa.edu.au; Tel.: +61-8-6488-4467

Supplementary materials

Table S1. Maternal and infant characteristics based on maternal secretor status.

Characteristics	Cohort (*n* = 60) Mean ± SD (Min-Max) or n (%)	Secretor (*n* = 49) Mean ± SD (Min-Max) or n (%)	Non-Secretor (*n* = 11) Mean ± SD (Min-Max) or n (%)	*p*-Value
Maternal characteristics				
Age at infant birth (years)	32.9 ± 4.7 (25.1–46.4)	32.8 ± 5.0 (25.1–46.4)	33.3 ± 3.0 (30.0–40.8)	0.66
Ethnicity				
Caucasian	51 (85)	41 (86.7)	10 (90.9)	-
Asian	5 (8.3)	5 (10.2)	-	-
Other	4 (6.6)	3 (6.1)	1 (9.1)	-
Parity	2.1 ± 0.8 (1–4)	1.9 ± 0.8 (1–4)	2.5 ± 0.7 (2–4)	0.06
Mode of delivery				0.07
Vaginal	38 (63.3)	28 (51.1)	10 (90.9)	
Elective caesarean section	14 (23.3)	14 (28.6)	-	-
Emergency caesarean section	8 (13.3)	7 (14.3)	1 (9.1)	-
Height (cm)	164.9 ± 6.7 (148.0–178.0)	164.5 ± 6.7 (148.0–178.0)	166.4 ± 7.2 (154.0–178.0)	0.45
Weight (kg)	72.3 ± 15.1 (47.5–119.8) [a]	72.0 ± 16.3 (47.5–119.8) [d]	73.8 ± 7.2 (63.1–86.6) [f]	0.60
Infant characteristics				
Gestational age (weeks)	39.3 ± 1.2 (37.0–41.2)	39.3 ± 1.2 (37.0–41.2)	39.3 ± 1.1 (37.4–41.1)	0.85
Male	29 (48.3)	23 (46.9)	6 (54.5)	-
Female	31 (51.7)	26 (53.1)	5 (45.5)	-

Birth weight (kg)	3.479 ± 0.432 (2.363–4.705)	3.488 ± 0.436 (2.363–4.705)	3.438 ± 0.432 (2.815–4.310)	0.73
Birth length (cm)	50.9 ± 2.4 (46.0–59.0)	50.9 ± 2.4 (46.0–59.0)	50.6 ± 2.1 (46.0–53.5)	0.64
Weight (kg)	6.161 ± 0.763 (4.638–8.410) [a]	6.116 ± 0.719 (4.638–8.204) [d]	6.365 ± 0.951 (5.038–8.410) f	0.45
Length (cm)	61.1 ± 2.6 (55.5–67.0) [a]	60.9 ± 2.5 (55.5–67.0) [d]	62.1 ± 2.7 (58.5–67.0) [f]	0.22
Head circumference (cm)	40.7 ± 1.2 (37.5–43.0) [b]	40.6 ± 1.3 (37.5–43.0) [e]	41.0 ± 1.1 (39.2–42.5) [g]	0.37
24-hour milk intake (mL)	784.7 ± 171.7 (511.7–1304.9) [c]	788.2 ± 179.1 (511.7–1304.9) [f]	767.6 ± 137.9 (561.2–999.0) [h]	0.72

Data are mean ± SD and ranges or percentage. All anthropometrics were measured at 3 months' postpartum unless otherwise stated. *p*-value describe the difference between secretor and non-secretor mothers (Welch's t-test for continuous maternal and infant characteristics and fisher's exact test for parity and mode of delivery) and significant *p*-values are shown as bold text. [a] $n = 55$, [b] $n = 50$, [c] $n = 47$, [d] $n = 45$, [e] $n = 40$, [f] $n = 39$, [g] $n = 10$, [h] $n = 8$.

Table S2. Maternal and infant body composition at 3 months' postpartum.

**Body Composition**	**Cohort (*n* = 55)**	**Secretor (*n* = 45)**	**Non-Secretor (*n* = 10)**
	Maternal		
Fat-free mass (kg) *	3.81 (0.16) (18.0–70.89)	3.80 (0.22) (18–70.89)	3.84 (0.02) (44.16–51.96)
Fat-free mass index (kg/m²) *	2.82 (0.17) (6.30–23.96)	2.81 (0.17) (6.3–23.96)	2.85 (0.12) (14.93–19.54)
Fat mass (kg) *	3.20 (0.40) (11.25–53.93)	3.19 (0.40) (11.25–53.93)	3.25 (0.25) (17.78–40.49)
Fat mass index (kg/m²) *	2.18 (0.41) (3.94–18.99)	2.16 (0.40) (3.94–18.99)	2.19 (0.39) (6.45–16.02)
Fat mass (%)	35.79 ± 6.01 (21.88–53.35)	35.74 ± 6.15 (21.88–53.35)	36.04 ± 5.6 (28.18–46.75)
Fat mass to fat-free mass ratio *	−0.63 (0.33) (0.37–1.14)	−0.63 (0.31) (0.37–1.14)	−0.63 (0.30) (0.39–0.88)
Body mass index (kg/m²)	26.63 ± 5.18 (18.00–40.95)	26.5 ± 5.41 (18–40.95)	27.23 ± 4.18 (21.57–34.25)
	Infant		
Fat-free mass (kg)	4.59 ± 0.52 (3.8–6.01) [a]	4.56 ± 0.51 (3.8–6.01) [c]	4.69 ± 0.59 (3.88–5.91)
Fat-free mass index (kg/m²)	14.98 ± 1.27 (12.93–18.52) [a]	14.96 ± 1.26 (12.93–18.52) [c]	15.1 ± 1.34 (13.25–17.63)
Fat mass (kg)	1.57 ± 0.3 (0.84–2.50) [a]	1.54 ± 0.29 (0.84–2.2) [c]	1.67 ± 0.37 (1.16–2.5)
Fat mass index (kg/m²)	5.1 ± 0.85 (3.02–7.47) [a]	5.05 ± 0.82 (3.02–6.76) [c]	5.35 ± 0.98 (3.97–7.47)
Fat mass (%)	25.3 ± 2.49 (17.11–29.76) [a]	25.14 ± 2.57 (17.11–29.04) [c]	26.01 ± 2.08 (22.7–29.76)
Fat mass to fat-free mass ratio	0.34 ± 0.04 (0.21–0.42) [a]	0.34 ± 0.04 (0.21–0.41) [c]	0.35 ± 0.04 (0.29–0.42)
Body mass index (kg/m²)	16.47 ± 1.29 (14.05–19.92)	16.47 ± 1.3 (14.05–19.92)	16.45 ± 1.31 (14.68–18.73)

Weight-for-length z-score	−0.09 ± 0.88 (−1.82–2.54)	−0.07 ± 0.92 (−1.82–2.54)	−0.18 ± 0.74 (−1.47–1.00)
Weight-for-age z-score	0.02 ± 0.89 (−2.79–2.49)	−0.03 ± 0.85 (−2.79–2.2)	0.23 ± 1.04 (−1.13–2.49)
Length-for-age z-score	0.22 ± 1.17 (−3.09–2.83)	0.13 ± 1.15 (−3.09–2.74)	0.62 ± 1.27 (−1.36–2.83)
BMI-for-age z-score	−0.09 ± 0.82 (−1.63–1.95)	−0.08 ± 0.84 (−1.63–1.95)	−0.15 ± 0.75 (−1.14–1.23)
Head circumference-for-age z-score	0.54 ± 0.99 (−2.47–2.56) [b]	0.49 ± 1.02 (−2.47–2.56) [d]	0.74 ± 0.91 (−1.13–1.76)

Data are mean ± SD and ranges. * Data log transformed and presented as median (interquartile range) and ranges. [a] n = 53, [b] n = 50, [c] n = 43, [d] n = 40. BMI – body mass index.

Table S4. Concentrations of the HMOs in human milk samples.

HMO	Cohort (n = 60)	Secretor (n = 49)	Non-Secretor (n = 11)	p-Value
2′FL	1517.97 ± 952 (0.29–3865.53)	1854.43 ± 696.13 (676.0–3865.53)	19.18 ± 57.19 (0.29–191.46)	**<0.001**
3FL *	7.38 (0.77) (132.64–2493.99)	7.48 (0.49) (132.64–1506.81)	0.17 (1.36) (461.38–2493.99)	**<0.001**
DFLac *	5.25 (0.81) (0.84–1043.33)	5.32 (0.60) (71.52–1043.33)	1.45 (0.82) (0.84–72.14)	**<0.001**
3′SL	88.49 ± 33.54 (10.25–211.85)	92.03 ± 33.54 (28.32–211.84)	73.80 ± 30.61 (10.25–125.67)	0.10
6′SL	149.14 ± 63.07 (23.49–369.20)	153.11 ± 62.80 (35.02–369.20)	131.50 ± 64.18 (23.49–234.27)	0.33
LNT *	6.35 (0.70 (202.88–1571.33)	6.33 (0.53) (202.88–1565.09)	6.95 (0.62) (472.84–1571.33)	**0.006**
LNnT *	4.37 (0.94) (6.79–245.68)	4.57 (0.76) (29.92–245.68)	3.51 (0.65) (6.79–74.97)	**<0.001**
LNFP I *	5.68 (1.28) (67.08–1671.46)	5.98 (1.19) (94.07–1671.46)	4.64 (0.50) (67.08–165.66)	**<0.001**
LNFP II *	6.40 (0.56) (194.60–1795.52)	6.33 (0.45) (194.60–1180.69)	7.19 (0.54) (436.74–1795.52)	**<0.001**
LNFP III *	2.21 (0.68) (1.83–23.87)	2.26 (0.68) (2.84–23.87)	2.08 (0.55) (1.83–21.32)	0.34
LSTb *	4.18 (0.57) (15.90–154.02)	4.10 (0.57) (15.90–154.02)	4.51 (0.35) (38.01–146.42)	**0.003**
LSTc *	4.06 (0.74) (10.87–156.86)	4.16 (0.67) (20.52–156.86)	3.58 (0.73) (10.87–73.31)	**<0.004**
DFLNT	722.41 ± 362.42 (7.54–1680.79)	811.0 ± 334.26 (7.54–1680.79)	327.76 ± 169.97 (25.91–633.51)	**<0.001**
LNH *	3.98 (0.71) (14.87–112.50)	4.02 (0.55) (14.87–112.50)	3.77 (0.51) (20.79–78.08)	0.07
DSLNT	123.99 ± 45.61 (39.09–254.36)	123.40 ± 46.94 (39.09–254.36)	126.62 ± 41.10 (51.92–195.20)	0.822
FLNH	200.56 ± 82.17 (57.98–422.69)	200.01 ± 82.0 (57.98–422.69)	202.97 ± 86.93 (85.14–343.37)	0.919
DFLNH *	4.40 (1.39) (6.42–419.15)	4.23 (1.28) (6.42–419.15)	4.82 (0.70) (33.08–193.06)	**0.027**
FDSLNH *	5.43 (1.01) (5.52–916.90)	5.06 (1.12) (5.52–690.77)	6.29 (0.59) (175.67–916.90)	**<0.001**

DSLNH	119.11 ± 52.09 (31.50–235.36)	120.50 ± 51.43 (31.50–235.36)	112.90 ± 57.10 (38.17–196.32)	0.691

Data are mean ± SD and ranges. * Data were log transformed and presented as median (interquartile range) and ranges. Concentrations are in µg/mL. *p*-value describes the difference between secretor and non-secretor groups (Welch's t-test) and significant *p*-values are shown in bold text. 2′FL – 2′-fucosyllactose; 3′SL – 3′-sialyllactose; 3FL – 3-fucosyllactose; 6′SL – 6′-sialyllactose; DFLac – difucosyllactose; DFLNH – difucosyllacto-N-hexaose; DFLNT – difucosyllacto-N-tetrose; DSLNH – disialyllacto-N-hexaose; DSLNT – disialyllacto-N-tetraose; FDSLNH – fucodisialyllacto-N-hexaose; FLNH – fucosyllacto-N-hexaose; LNFP I – lacto-N-fucopentaose; LNFP II – lacto-N-fucopentaose II; LNFP III – lacto-N-fucopentaose; LNH – lacto-N-hexaose; LNnT – lacto-N-neotetraose; LNT – lacto-N-tetrose; LSTb – sialyl-lacto-N-tetraose b; LSTc – sialyl-lacto-N-tetraose c.

Table S5. 24-hour intakes of the HMOs.

HMO	Cohort (n = 47)	Secretor (n = 38)	Non-Secretor (n = 9)	*p*-Value
2'FL	1206.44 ± 728.09 (0.22–2729.05)	1449.76 ± 533.09 (524.63–2729.05	20.28 ± 54.90 (0.22–156.14)	**<0.001**
3FL	669.81 ± 417.99 (134.97–1820.51)	533.36 ± 322.60 (134.97–1436.59)	1237.49 ± 373.29 (688.47–1820.51)	**0.001**
DFLac *	4.98 (0.81) (0.71–1057.51	5.08 (0.69) (43.47–1057.51)	1.13 (0.67) (0.71–58.84)	**<0.001**
3'SL *	4.25 (0.47) (8.36–194.26)	4.28 (0.48) (30.45–194.26)	3.95 (0.35) (8.36–79.30)	0.084
6'SL	114.15 ± 47.20 (23.46–263.46)	118.25 ± 46.49 (33.99–263.46)	94.14 ± 48.54 (23.46–160.92)	0.23
LNT *	6.12 (0.63) (161.70–1117.23)	6.11 (0.60) (161.70–1030.59)	6.62 (0.86) (265.34–1117.23)	0.073
LNnT	69.28 ± 37.66 (6.78–155.75)	77.99 ± 34.92 (22.56–155.75)	26.80 ± 14.62 (6.78–50.97)	**<0.001**
LNFP I *	5.46 (1.02) (37.64–1092.25)	5.60 (0.88) (67.13–1092.25)	4.45 (0.72) (37.64–157.0)	**<0.001**
LNFP II *	6.01 (0.69) (170.63–1383.07)	5.96 (0.55) (170.63–978.65)	6.88 (0.62) (506.55–1383.07)	**<0.001**
LNFP III	7.47 ± 3.42 (1.42–17.73)	7.76 ± 3.45 (2.03–17.73)	6.09 ± 3.08 (1.42–9.36)	0.200
LSTb *	3.86 (0.79) (11.35–133.04)	3.79 (0.74) (11.35–114.40)	4.26 (0.39) (29.52–133.04)	**0.033**
LSTc	51.40 ± 27.43 (6.10–122.14)	56.42 ± 26.75 (21.17–122.14)	26.91 ± 15.32 (6.10–58.93)	**<0.001**
DFLNT	597.64 ± 333.62 (4.58–1605.39)	667.31 ± 320.21 (4.58–1605.39)	257.97 ± 126.18 (122.26–505.09)	**<0.001**
LNH *	3.70 (0.45) (11.6–143.20)	3.79 (0.56) (19.76–143.20)	3.38 (0.40) (11.66–48.54)	**0.017**
DSLNT	97.22–40.96 (27.89–193.28)	96.81 ± 40.29 (27.89–193.28)	99.18 ± 46.97 (40.33–167.12)	0.897
FLNH *	4.98 (0.52) (53.88–396.43)	5.01 (0.46) (53.88–396.43)	4.79 (0.65) (69.43–239.49)	0.42
DFLNH *	4.18 (1.68) (7.68–276.14)	3.86 (1.69) (7.68–276.14)	4.49 (0.83) (35.20–167.26)	**0.04**
FDSLNH *	5.14 (1.32) (3.50–769.32)	4.76 (1.21) (3.50–769.32)	5.83 (0.32) (280.46–653.57)	**<0.001**
DSLNH *	4.42 (0.80) (21.42–185.06)	4.45 (0.72) (24.16–185.06)	4.18 (1.02) (21.42–171.53)	0.503

Data are mean ± SD and ranges. * Data log transformed and presented as median (interquartile range) and ranges. HMOs intakes are measured as µg/L. *p*-value describes the difference between secretor and non-secretor groups (Welch's t-test) and significant *p*-values are shown in bold text. 2′FL – 2′-fucosyllactose; 3′SL – 3′-sialyllactose; 3FL – 3-fucosyllactose; 6′SL – 6′-sialyllactose; DFLac – difucosyllactose; DFLNH – difucosyllacto-N-hexaose; DFLNT – difucosyllacto-N-tetrose; DSLNH – disialyllacto-N-hexaose; DSLNT – disialyllacto-N-tetraose; FDSLNH – fucodisialyllacto-N-hexaose; FLNH – fucosyllacto-N-hexaose; LNFP I – lacto-N-fucopentaose; LNFP II – lacto-N-fucopentaose II; LNFP III – lacto-N-fucopentaose; LNH – lacto-N-hexaose; LNnT – lacto-N-neotetraose; LNT – lacto-N-tetrose; LSTb – sialyl-lacto-N-tetraose b; LSTc – sialyl-lacto-N-tetraose c.

Table S6. Associations of parity and mode of delivery with human milk components and infant body composition.

Predictor	Non-Secretor						Secretor			Interaction
	**Intercept**	**SE**	***p*-Value**	**Parameter Value**	**SE**	***p*-Value**	**Parameter Value**	**SE**	***p*-Value**	***p*-Value**
	HMO concentration									
	Log (LNFP III)									
Parity (2)	2.4661	0.2642	<0.001	−0.86	0.32	**0.01**	−0.26	0.15	0.10	0.06
	Human milk bacteria									
	OTU05 (*Streptococcus parasanguis*)									
MOD (ECS)	−0.84	0.77	0.28	−1.85	1.26	0.15	2.53	0.93	**0.01**	<0.001
	Infant body composition									
	Weight (kg)									
MOD (ECS)	6.2119	0.3523	<0.001	2.20	0.80	**0.01**	−0.26	0.35	0.46	0.004
	Fat mass (kg)									
MOD (ECS)	1.6419	0.1398	<0.001	0.86	0.32	**0.01**	−0.10	0.14	0.46	0.005
	Log fat-free mass (kg)									
MOD (ECS)	1.5168	0.0534	<0.001	0.26	0.12	**0.04**	−0.03	0.05	0.52	0.02
	Fat mass index (kg/m^2)									
MOD (ECS)	5.3269	0.3994	<0.001	2.14	0.90	**0.02**	−0.33	0.39	0.40	0.01
	Cohort									
	HMO concentrations									
	Log (LNFP II)									
Parity (3+)	6.856	0.165	<0.001	0.30	0.14	**0.03**				
	Log (FDSLNH)									
MOD (ECS)	5.6535	0.3631	<0.001	1.08	0.37	**0.005**				

Data are parameter value ± standard error of measurement (SE). ANOVA was fit for parity and mode of delivery. Significant *p*-values indicated by the bold text. ECS – emergency caesarean section; FDSLNH – fucodisialyllacto-N-hexaose; LNFP II – lacto-N-fucopentaose II and LNFP III – lacto-N-fucopentaose III; MOD – mode of delivery.

Table S7. Summary of OTUs detected in negative extraction controls (n = 2) and negative PCR controls (n = 2).

OTUs	Genus	Extraction Control 1	Extraction Control 2	PCR Control 1	PCR Control 2
OTU20	*Burkholderia-Caballeronia-Paraburkholderia*		65		13089
OTU29	*Allorhizobium-Neorhizobium-Pararhizobium-Rhizobium*		27		7083
OTU17	*Vibrionimonas*	15617			453
OTU40	*Schlegelella*	823			23
OTU106	*Vibrionimonas*	507			15

OTU01	*Streptococcus*	7	14	7	9
OTU23	*Bradyrhizobium*	246			7
OTU39	*Lawsonella*			1147	
OTU515	*Vibrionimonas*	11			
OTU03	*Streptococcus*		6		
Others	Others	298	26	25	316

Only OTUs forming more than 5 reads in controls are presented. OTU – operational taxonomic unit; PCR – polymerase chain reaction.
